# Targeting Cellular Metabolism Modulates Head and Neck Oncogenesis

**DOI:** 10.3390/ijms20163960

**Published:** 2019-08-14

**Authors:** Yi-Ta Hsieh, Yi-Fen Chen, Shu-Chun Lin, Kuo-Wei Chang, Wan-Chun Li

**Affiliations:** 1 Institute of Oral Biology, School of Dentistry, National Yang-Ming University, Taipei 11221, Taiwan; 2 Department of Dentistry, School of Dentistry, National Yang-Ming University, Taipei 11221, Taiwan; 3 Department of Stomatology, Taipei Veterans General Hospital, Taipei 11217, Taiwan; 4 Cancer Progression Research Center, National Yang-Ming University, Taipei 11221, Taiwan

**Keywords:** head and neck cancer, metabolic reprogramming, tumor microenvironment, non-coding RNA, targeted therapy

## Abstract

Considering the great energy and biomass demand for cell survival, cancer cells exhibit unique metabolic signatures compared to normal cells. Head and neck squamous cell carcinoma (HNSCC) is one of the most prevalent neoplasms worldwide. Recent findings have shown that environmental challenges, as well as intrinsic metabolic manipulations, could modulate HNSCC experimentally and serve as clinic prognostic indicators, suggesting that a better understanding of dynamic metabolic changes during HNSCC development could be of great benefit for developing adjuvant anti-cancer schemes other than conventional therapies. However, the following questions are still poorly understood: (i) how does metabolic reprogramming occur during HNSCC development? (ii) how does the tumorous milieu contribute to HNSCC tumourigenesis? and (iii) at the molecular level, how do various metabolic cues interact with each other to control the oncogenicity and therapeutic sensitivity of HNSCC? In this review article, the regulatory roles of different metabolic pathways in HNSCC and its microenvironment in controlling the malignancy are therefore discussed in the hope of providing a systemic overview regarding what we knew and how cancer metabolism could be translated for the development of anti-cancer therapeutic reagents.

## 1. Introduction

Malignancies of the head and neck influence a variety of anatomic sites, including the oral cavity, oropharynx, nasopharynx, hypopharynx, larynx, and salivary glands [1]. The oncogenic stimuli of Head and Neck Squamous Cell Carcinomas (HNSCC), including smoking, alcohol consumption, viral infection and an imbalanced metabolism, could lead to genetic mutations and epigenetic modulations that serve as potential triggers for head and neck tumorigenesis [2,3]. Clinical therapeutic regimens for HNSCC patients have been widely discussed; single or combinational treatments of surgery, chemotherapy and radiotherapy are common choices for HNSCC, depending on the tumor sizes, locations, histological subtypes and clinical stages [4,5]. Nevertheless, 5-year survival rates for HNSCC patients are still below 50% and have not changed much over the past 50 years. The poor survival rates could be attributable to the late diagnosis of the disease, lack of better prognostic tools or development of resistance to conventional therapies [6,7,8,9,10].

**Figure 1:**
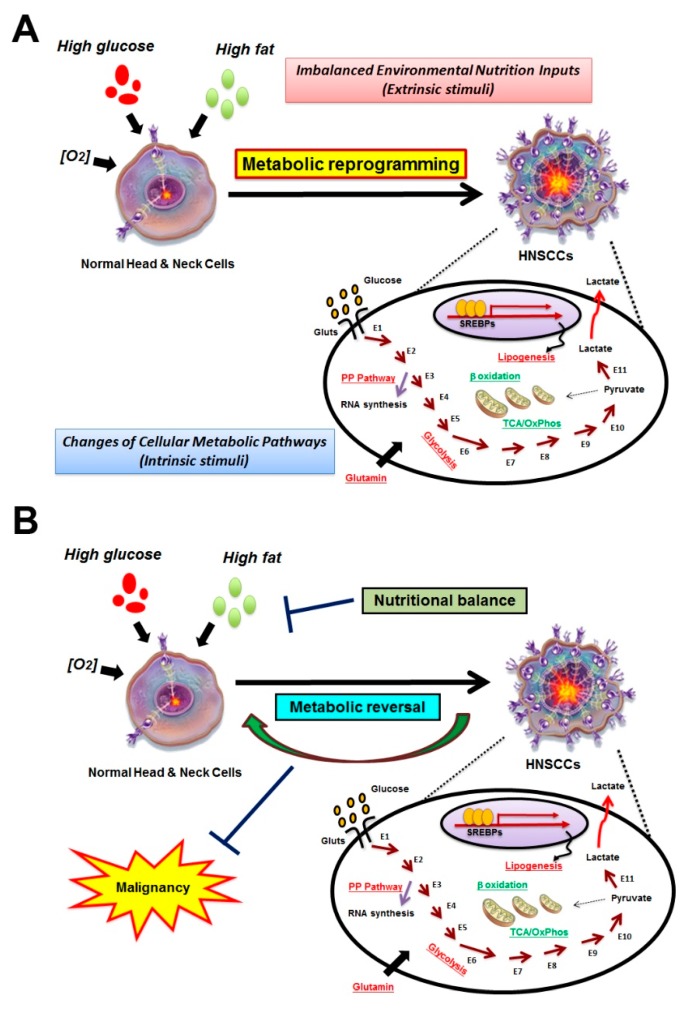
Metabolic reprogramming in HNSCCs. (**A**) Diagraphic illustration of the metabolic shift during the oncogenic transformation in HNSCC cells. The pathways presented in red indicate the pathways upregulated in HNSCC cells compared to normal cells, while the metabolic pathways shown in green are pathways less active in tumors; (**B**) the nutritional balance (e.g., maintenance of normoglycemia in DM patients) and reverse for intrinsic metabolic cues by inhibitors could be a potential method to suppress cancerous identity in HNSCCs. E1-E11: Enzymes in glycolytic pathway.

Although cancer is generally considered to be a genetic disease [11], inconsistencies regarding the somatic nuclear gene theory based on nuclear/cytoplasmic transfer experiments between tumorigenic and non-tumorigenic cells show that tumorigenicity could originate from disrupted metabolic homeostasis [12,13,14]. To meet great demands for cell growth, neoplastic cells require large quantities of energy and macromolecules from an extracellular milieu; the extrinsic signals could then be transduced into cells and co-opt the numbers of core metabolic pathways, including glycolysis, mitochondrial metabolism, and lipid and amino acid anabolism/catabolism to support cell survival [15,16,17]. On a physiological level, oxygen availability is important for cancer cells to determine their metabolic identities, as cells in tumor tissues expose to various oxygen levels with respect to their distance from the closest blood vessels [18] (Figure 1A). While cancer metabolism is receiving increasing attention [19,20], most studies were conducted to target a single metabolic enzyme or metabolite in controlling tumorigenesis, without analyzing global metabolic alterations. In this way, to escape death, cancer cells could possibly evolve and develop alternative compensatory metabolic changes [21]. In light of this, systemic manipulations to direct the tumor cell metabolic status “back” to the normal cell status, thereby lessening the cancer malignancy, is desired (Figure 1B). To achieve this aim, the identification of reagent(s) that could reduce preferential metabolic effectors in tumors as well as trigger unfavorable carcinogenic metabolic cues could be molecules of interest for suppressing malignancy in cancers. The goal of the review is to provide a systemic overview regarding the current understanding of cancer metabolism and its clinical potential, with an emphasis on HNSCCs.

## 2. Identification for HNSCC-Specific Metabolic Profile

In early years, HNSCC-specific metabolic fingerprints were defined mainly by immunohistochemistry staining analysis and serological examination. For example, cellular retinoic acid binding protein (CRABP) expression was enriched in tumor tissues compared with its adjacent normal tissues [22], while additional studies confirmed that external retinoic acid administration could modulate the Epidermal Growth Factor Receptor (EGFR) activity, a key predisposition of HNSCC development [23]. Furthermore, a higher glutathione (GSH) concentration was detected in metastatic tumors, compared with those concentrations derived from the corresponding primary lesions, suggesting a possible impact of the GSH metabolism on the formation of metastases in HNSCCs [24,25]. Indeed, more recent studies determined prognostic roles of glutathione metabolic enzymes such as glutathione-S-transferases in controlling HNSCC oncogenicity [26]. Other investigations focusing on the association of the ornithine decarboxylase activity with cellular DNA distributions [27], the influence of the intracellular cAMP:cGMP ratio on protein kinase activity and cell growth [28] and the impact of decreased polyglutamylation for methotrexate resistance [29], were also described. On the other hand, the results showing that the serological metabolite orosomucoid:prealbumin ratio predicted a clinical prognosis [30] and total parenteral nutrition enhanced cell susceptibility to the cell cycle inhibitor [31] further confirmed that circulating metabolic molecules could affect the HNSCC malignancy. Interestingly, a study led by Byerley et al. showed that, using a computerized euglycemic clamp, a significantly lower plasma insulin concentration and elevated whole body glucose appearance were detected in cancer patients than in control subjects, indicating that tumors could act as a glucose drain, aiding against local cachexia [32].

## 3. Association of Systemic Disease and HNSCCs

From a broad point of view, the correlation between systemic diseases and HNSCC cancer is an interesting topic worthy of further investigations. Taking Diabetes mellitus (DM) as an example, although for more than fifty years clinicians have reported that DM and cancer are diagnosed within the same individual more frequently, both diseases are complex with multiple subtypes, making it difficult to conclude the association between them [33]. Previous epidemiological reports aiming to clarify the association between DM and oral pre-cancerous malignancy or cancer are sparse and highly debated as the conclusions are rather inconsistent. The relationship between DM and pre-cancerous lesions was more emphasized. A very early review examining 1600 DM patients for the occurrence of oral leukoplakia (OL) and oral lichen planus (OLP) found that the prevalence of OL and OLP in DM patients was higher compared to healthy controls [34]. It was also reported that DM is a significant and independent predictor of OL [35], while the results from the population-based Study of Health in Pomerania (SHIP) showed that subjects with OL exhibited higher levels of DM-related metabolites, suggesting that DM is associated with the risk of OL [36]. A recent cohort study with a samples size of more than 4.5 million US veterans reported a significantly low risk of Oral Squamous Cell Carcinomas (OSCCs) in DM patients [37]. However, this study recruited only male patients, and the age of the groups are rather old (> 50 years old), limiting further interpretation of the results. Local studies from Taiwanese hospitals brought about different conclusions regarding the role of DM in correlating with oral carcinogenesis or prognosis. It was reported that DM patients tend to have a lower overall survival, recurrence-free survival and cancer-specific survival compared to non DM subjects, even in less aggressive tumor stages (stages I and II) [38]. Nevertheless, a recent epidemiological study showed that the DM status and duration were strongly associated with an OSCC prevalence in unadjusted models but that none was significant after a multivariable adjustment [39]. At the molecular level, using an animal model, it was demonstrated that insulin receptor substrat-1 (IRS-1) and focal adhesion kinase might be potential molecules leading to an increased DM-mediated risk of oral cancer development [40]. Our group recently demonstrated that prolonged diabetic/hyperglycemic incubation is significantly correlated with phenotypic aggressiveness in HNSCC cells in vitro, in vivo and in clinic, further implying that DM is likely a positive regulator for HNSCC/OSCC development [41]. In brief, external metabolic challenges could modulate HNSCC oncogenicity; however, it is still less feasible to define the cause-and-effect relationship between systemic diseases such as DM and HNSCC/OSCC development, particularly in a clinical setting, since both diseases are age-related.

## 4. Development of Anti-Cancer Scheme by Targeting Distinct Metabolic Pathways in HNSCCs

As discussed in recent studies, by using NMR spectroscopy, HNSCC-specific metabolic features correlated with an acquired EGFR-TKI resistance [42]; the intracellular metabolism therefore becomes one of the potential targets to develop non-canonical anti-HNSCC drugs to better improve therapeutic efficiency. Below, we therefore focus in detail on studies seeking the development of metabolism-mediated anti-HNSCC scheme(s)/molecule(s) by targeting different pathways.

### 4.1. Targeting Aerobic Glycolysis in HNSCCs

Hyperactive glycolytic metabolism is the most promising metabolic change in highly proliferating cells [43]. Despite a lesser amount of energy production, many rapidly growing cells rely primarily on glucose fermentation during proliferation, regardless of oxygen availability, a phenomenon known as aerobic glycolysis or the “Warburg effect” [44]. Aerobic glycolysis is a metabolic hallmark uniquely observed in many cancers and had therefore been considered a well-studied target for developing cancer sensors or inhibitors [45]. For example, 2-deoxy-D-glucose (2-DG), a glucose analogue, has been used as medical detector in Positron Emission Tomography (PET) scanning to detect tumors in clinic [46,47], including HNSCCs [48]. Other studies also found that the combinational treatment of 2-DG with cisplatin (CDDP) and metformin in HNSCC cells exhibited an improved therapeutic efficacy compared with a single treatment, through the enhancement of oxidative stress, both in vitro and in vivo [49,50,51]. Moreover, the suppression of the Glucose transporter SLC2A (so-called GLUT-1) activity by the selective inhibitors Fasentin and WZB-117 decreased the HNSCC malignancy [52,53]. Other anti-cancer glycolytic enzyme inhibitors in HNSCC cells were widely studied. For example, the HK II inhibitor Fenofibrate, acting through the suppression of the binding of HK II and the mitochondrial transporter VDAC, modulates the AKT/mTOR signaling pathway and was found to repress tongue tumor development [54]. The PFKFB3 inhibitor PFK15 could induce cancer cell apoptosis, via the reduction of glucose uptake and lactate production [55]. The LDHA inhibitor Oxamate, acting via direct targeting of LDHA activity by suppressing a conversion of pyruvate to lactate, was reported to suppress cell growth in cetuximab (EGFR inhibitor) resistance HNSCC cells in vitro [56] or to inhibit tumor growth in vivo [57]. In agreement with previous findings, our group also recently defined LDHA as an oncogenic cue in regulating HNSCC malignancy in vitro, in vivo and in clinic [21]. At the molecular level, another interesting finding showed that the inhibition of the membrane bidirectional transporter MCT1, which converts monocarboxylates such as lactate into pyruvate for more energy supply (in HNSCC derived xerographic tumors, via AZD3965 (in combination with a simvastatin treatment)), could delay tumor growth [58,59]. Taken together, this confirms that targeting glycolytic molecules could efficiently suppress tumor growth — even though to date no glycolytic inhibitors are used as anti-HNSCC agents in clinic. One possibility for this slow progress might be due to the fact that it is still difficult to clearly define the differential dependency of glycolysis in HNSCC tissues compared with non-cancerous tissues in vivo. On a cellular level, how HNSCC cells stimulate alternative compensatory non-glycolytic metabolic changes to evade cell death in response to the treatment of glycolytic inhibitors remains to be determined.

### 4.2. Targeting Mitochondrial Related Metabolism in HNSCCs

The progression of biogenetic changes during cancer development, based on Warburg’s theory, is: insufficient respiration acts early during malignant transformation, followed by a compensatory energy production through glycolysis to continuously ferment lactate in the presence of oxygen, leading to deficient oxidative phosphorylation (OxPhos) that is irreversible [14,44]. Indeed, a number of mitochondrial transfer experiments support this statement by demonstrating that various tumorigenic phenotypes could be suppressed when mitochondria from normal cells are transferred to a tumor cell, whereas the tumorigenicity is enhanced when tumor mitochondria are transferred into a normal cell [60,61]. Furthermore, in contrast to normal mitochondria, which contain numerous cristae, structures with respiratory proteins that play essential structural roles in facilitating energy production through OxPhos, mitochondria from tumor tissues showed swelling cristae with partial or total cristolysis, implying that a respiratory defect is indeed one of the key hallmarks of cancer cells [62]. The numbers of metabolic pathways related to ATP production, including the TCA cycle and Electron Transport Chain (ETC) reactions, occur within mitochondria. According to Warburg’s observation, tumor cells avidly used aerobic glycolysis, as mitochondrial respiration exhibits an irreversible defect, suggesting that mitochondrial dysfunction might also play a key role in controlling malignant transformation. Thus, a further emphasis on how mitochondrial metabolic cues, including the ROS-mediated apoptotic machinery, TCA cycle and ETC, regulate the HNSCC malignancy and are, furthermore, targets for the development of an anti-HNSCC therapeutic method is discussed [63,64,65].

#### 4.2.1. Reactive Oxygen Species (ROS) and Apoptotic Pathway

ROS, a by-product of ETC, are molecules that could capture a singlet oxygen atom in mitochondria under a normal physiological condition. Under a certain pathological status, an accumulating ROS generation results in an imbalanced respiration chain, thereby leading to oxidative stress, a well-known toxic physiological stimulus for various diseases including cancer formation [66,67]. Providing the importance of ROS in tumorigenesis, a number of studies have been reported to target the ROS level with some natural compound like Flavonoid or Triterpene, which was extracted from a plant or fruit, aiming to establish alternative approaches to lessen the HNSCC malignancy. In general, these agents could induce a decrease of the mitochondrial activity, thereby reducing the HNSCC cell viability — although different mechanisms were proposed. For example, Hederagenin inhibits the NRF2/ARE mediated antioxidant pathway, thereby increasing the ROS production in OSCC cells [68]. CHW09 and CYT-Rx20, two novel chemical compounds, also trigger the ROS levels and mitochondrial superoxide (MitoSox), followed by Glutathione suppression [69,70]. Carbolitin is a less toxic analog of CDDP, which when combined with Thioridazine has a dramatic decrease on cell proliferation due to the upregulation of proteasome subunit alpha 5 (PSMA5) via ROS production [71]. Interestingly, the treatment of Bufalin triggers cell growth inhibition via an ROS-independent mechanism based on the detection of an increased nitric oxide level and calcium production, as well as decreased ROS levels and Mitochondrial Membrane Potential (MMP) in HNSCCs [72]. In addition to the ROS-mediated cell death pathway, other studies also suggested that mitochondrial dysfunction could also modulate HNSCC growth. For example, Fisetin could induce cytochrome C, an apoptosis-inducing factor, as well as Endonuclease G, released from mitochondria into the cytosol and could trigger an apoptotic pathway [73]. Casticin treatment led to cell death via a G2/M cell cycle arrest [74]. Moreover, HL156A, a derivative of metformin, could inhibit the oncogenic Akt-mTOR-ERK1/2 pathway, thereby downregulating cancerous phenotypes in OSCCs [75]. In brief, chemical compounds that could facilitate mitochondrial-mediated cell death pathways could be ideal targets for the development of anti-HNSCC drugs, although the effects on normal cells need to be evaluated before clinic use.

#### 4.2.2. Tricarboxylic Acid Cycle (TCA Cycle) & Electron Transport Chain (ETC)

It is widely known that the TCA cycle acts as an essential metabolic pathway for the cell energy supply. The numbers of compounds were therefore developed to modulate the TCA cycle aiming to regulate cellular bioenergetics-mediated phenotypes in cancers. For HNSCCs, it was reported that Lanthanide could inhibit HNSCC growth in glucose free conditions by suppressing cytosolic Malic enzyme 1 (ME1) that converts malate into pyruvate, leading to a decreased pyruvate level and consequent lactate production, accompanied by increased mitochondrial areas. This study suggested a metabolic shift away from aerobic glycolysis toward oxidative phosphorylation in response to lanthanide treatment [76]. A more recent study further demonstrated that ME2 depletion potentiated ionizing radiation therapy induced cellular senescence driven by ROS and linked to better clinical outcomes, revealing a potential therapeutic benefit of targeting ME2 [77]. Moreover, rotenone, a specific inhibitor for ETC complex I, has been shown to induce apoptosis in HNSCC via the activation of the caspase 8/9 mediated pathway [78]. It was recently found that a common anti-diabetic drug, metformin, could reduce the mitochondrial respiration activity and increase the sensitivity to external-beam radiation (XRT) in HNSCC carrying the wild-type TP53 protein, under a combinational treatment of 2-DG [49,79]. Another interesting finding was to discover the unexpected mitochondrial regulatory impact of “old” drugs, showing that atovaquone, an anti-malarial drug, inhibited complex III activity and caused an OCR reduction, leading to a reduced tumor growth in HNSCCs [80].

At a genetic basis, mitochondria possess their own transcriptional codes. Mitochondrial DNA (mtDNA) is located in the mitochondrial matrix and encodes 37 subunits of the tETC complex involved in the OxPhos system. Based on previous findings, HNSCC cells utilize aerobic glycolysis preferentially over the mitochondrial metabolism, suggesting that a decreased OxPhos activity is a potential predisposition for HNSCC development [21,81]. Indeed, it was found that a significantly lower mtDNA copy number was detected in the peripheral blood leukocytes of oral premalignant lesion patients when compared to those from a healthy group [82]. In HNSCC patients, lower COX II mRNA as well as D-loop abundance was detected in cancerous tissues when compared with their normal counterparts [83]. Consistent with these results, by using the TCGA database, less mtDNA content is found in HNSCC tumors in contrast to adjacent normal tissues [84]. Nevertheless, another study found that COX I mRNA expression was upregulated during HNSCC development from a benign lesion to a premalignant one, and ultimately to the tumorous stage [85], implying that the regulatory role of the mtDNA number is still unclear in HNSCCs. In brief, a number of questions remain to be elucidated; they include: (1) What are the upstream signaling pathway(s) for controlling the mtDNA expression? (2) Could a nuclear DNA-encoded subunit compensate for an mtDNA deficiency? (3) What are the associations between epidemiological risk factors including racism, smoking, alcohol consumption, areca nut chewing and HPV infection, and the mtDNA content in HNSCC patients? and (4) Is mtDNA modulation or OxPhos activity playing a more important role in regulating HNSCC oncogenicity?

### 4.3. Targeting Lipid Metabolism in HNSCCs

Cancer cell proliferation also requires the duplication of cellular macromolecular components during each cell division, making lipid metabolism a principal physiological pathway for maintaining cell architecture, growth and physiological homeostasis [86,87,88,89]. The link between glycolysis and the lipid metabolism could be evident by the fact that a portion of acetyl-CoA could be carboxylated into malonyl-CoA by acetyl-CoA carboxylase (ACACA), the primary rate-limiting enzyme of the de novo fatty acid biosynthesis pathway, followed by the condensation of acetyl-CoA and malonyl-CoA by fatty acid synthase (FASN) to produce saturated fatty acids (FAs). Saturated long-chain FAs can be further modified by elongases or desaturases to form more complex FAs, which are used for the synthesis of various cellular lipids such as phospholipids, triglycerides and cholesterol esters [90,91]. While FA building blocks come from either exogenous sources or from de novo biosynthesis, most normal cells prefer an external uptake, as tumors synthesize FA mainly through intrinsic de novo lipogenesis [92,93]. Based on previous studies, aberrant lipid metabolism is now recognized as one of the key features of cancer cells because cell proliferation requires an increased lipid biosynthesis in order to produce bioactive molecules that act as signal molecules regulating cancer progression [94]. Indeed, earlier studies showed that elevated activities of citrate synthase (CS), FASN and ATP citrate lyase (ACLY) were observed in different malignant cells [95,96]. The blockage of key lipid metabolic enzymes such as ACLY, FASN, ACACA, and stearoyl CoA desaturase (SCD), as well as the upstream regulator sterol regulatory element-binding proteins (SREBPs) in various neoplastic cells could suppress tumor cell malignancy both in vitro and in vivo [92,97,98,99]. We and other groups also found that either the imbalanced circulating cholesterol or upregulated expression of FASN, ACACA and SREBPs correlated with a poorer diagnosis in different cancers, implying a significance of the lipid metabolism during the cancer development in clinic [100,101,102].

As for HNSCC, the significance of the lipid metabolism in controlling the pathological transformation, in comparison with glycolysis- and mitochondria-related cues, is much less emphasized. It was firstly studied from the findings that cholesterol-lowering drugs, including lovastatin and simvastatin, exhibited a great anti-HNSCC effect by facilitating cell apoptosis and attenuating 1 integrin mediated cell migration, implying that circulating lipids might play a role in regulating HNSCC malignancy [103,104]. Further investigations revealed that elevated lipid peroxidation products such as lipid-derived ROS and Reactive Nitrogen Species (RNS) could be crucial in the HNSCC development [105,106,107], given that the antioxidant compound apigenin suppressed CDDP/5-fluorouracil (5-Fu) induced cytotoxicity via the Tumor Necrosis Factor (TNF)/Bcl-2 pathway [108]. The link between the lipid metabolism associated molecules and neoplastic phenotypic changes in HNSCC was initially evident from a finding that caspase-triggered SREBP activation is a key molecular mechanism for HNSCC cell death under CDDP treatment [109]. A number of studies were later conducted to delineate the roles of different lipogenic proteins, including ACCs [110,111,112], FASN [113] and sphinogosine-1-phosphate metabolic enzymes [114], as well as a lipid catabolic factor lipolysis-stimulated lipoprotein receptor (LSR) [115] in controlling the HNSCC malignancy. In general, the molecules involved in lipogenesis are upregulated during HNSCC development and serve as an oncogenic indicator in clinic [110,114,116]; in contrast, lipid catabolic cues display a tumor-suppressive effect in HNSCCs.

### 4.4. Targeting Amino Acid Metabolism in HNSCCs

Like glucose, there are major differences in the utilization and metabolism of amino acids in tumors when compared to normal cells. All twenty amino acids could serve differently as important regulatory cues to many of the physiological processes, including the biosynthesis of macromolecules, cellular redox homeostasis, post-translational and epigenetic modifications and anaplerostic reactions [117,118,119]. Previous studies have found that several amino acids, including phenylalanine, valine, threonine, tyrosine, glycine, proline, histidine, aspartic acid, glutamic acid, asparagine, lysine, serine, methionine, and alanine, were significantly increased in tumor tissues compared with adjacent normal tissues from HNSCC patients [120,121]. By using L-[3-18F]-α–methyltyrosine (18F-FAMT) as a PET imaging tracer, it was also discovered that primary tumor cells, as well as cervical lymph node tissues, exhibit a greater amino acid uptake rate in HNSCC patients, suggesting that the amino acid metabolism might serve a critical role in HNSCC oncogenicity [4].

In addition to intrinsic amino acids, cells could obtain amino acids externally via different transporters, including the L-type amino acid transporter 1 (LAT1), which is specific for the branched-chain and aromatic amino acid intake; the system ASC amino-acid transporter-2 (ASCT2), which is responsible for the neutral amino acid uptake; and SLC7A11, also named xCT, an bidirectional exchanger for cysteine-glutamate. Previous studies have found that these transporters are enriched in many cancers and that their expression is correlated with a worse survival rate [117,122,123,124,125,126,127,128]. In HNSCC tissues, a greater ALT expression positively correlated with a higher percentage of Ki67+ cells [129]. Further studies confirmed the role of LAT in controlling HNSCC tumorigenicity via the detection of the anti-proliferative ability in HNSCC cells, both in vitro and in vivo, in response to a combinational treatment of the LAT-specific inhibitor JPH203 and metformin [130]. The synergetic anti-cancer effect of LAT inhibition was also reported in a study using gefitinib, a clinically targeted therapeutic EGFR inhibitor, to enhance cytotoxicity under stress (e.g., amino acid starvation culture condition) in HNSCC cells [131]. Several studies have also documented that the other two amino acid transporters ASCT2 and xCT mediate chemotherapy and EGFR-targeted therapy resistance in HNSCC cells after treatments of CDDP, cetuximab and AG1478 [132,133,134], implying that amino acid transporters could be a therapeutic target in patients with HNSCCs. On a molecular basis, a recent study demonstrated that xCT-mediated therapeutic resistance occurs mainly through the regulation of ferroptosis [135].

Including different amino acids in the discussion, individual amino acids play differential roles in regulating tumor cell malignancy. For example, methionine-derivative S-adenosyl methionine (SAM) is a cofactor acting mainly as a methyl donor to cytosine in DNA during epigenetic modification [136]. A recent study found a reduced oral mucositis after an oral D-methionine treatment in patients with grade 3/4 HNSCCs [137]; an in vitro study showed that the administration of methionine could also downregulate the proliferative capacity in HNSCC cells; it was also reported that there was a positive correlation of high methionine serum levels with greater overall and relapse-free survival rates [121,138]. These findings suggest that methionine could be tumor-suppressive and that the methionine content might serve as a prognostic factor for HNSCC patients, although one must still mechanistically clarify the way in which methionine regulates epigenetic modification in HNSCC development. Another important amino acid for cell homeostasis is arginine. Arginine is a conditional essential amino acid that could either be obtained from a diet or from a biosynthesis pathway by converting glutamine into citrulline. Several studies showed that cell proliferation is abolished when HNSCC cells were cultured in an arginine-free medium or a medium containing an arginine depleting agent, Arginine deiminase (ADI) [139,140]. In addition, a disease-free survival and late-stage overall survival rates seem significantly correlated with the expression of argininosuccinate synthetase (ASS), a rate-limiting enzyme of the urea cycle that involves an arginine de novo synthesis by converting citrulline into argininosuccinate and arginine [139]. These findings revealed the oncogenic impact of the arginine-related metabolism and provide a possibility for targeting the arginine metabolic pathway for HNSCC therapy. A very recent investigation found that cysteine deficiency led to a decrease of the glutathione level, resulting in lipid ROS-mediated cell death in CDDP resistant HNSCC cells compared with their parental counterparts [135]. 

Glutaminolysis, a metabolic pathway for converting glutamine into glutamate via glutaminase, is widely documented as playing an essential role in regulating neoplastic phenotypes. Immunohistochemistry staining and Western blot analysis both showed a higher glutaminase expression in metastatic HNSCC tissues than in normal tissues. Furthermore, shRNA-mediated silencing for glutaminase abrogated the HNSCC proliferation and downregulated the cellular glutamate level. At the cellular level, a positive correlation between glutaminase and aldehyde dehydrogenase (ALDH) was reported, while the administration of glutamine could enhance the ALDH expression and restored the tumor growth in ALDHloCD44lo cells, suggesting that glutaminolysis-related manipulations could be a potential method for the development of a HNSCC stem cell based therapeutic regimen [141]. Another glutamine-related enzyme, glutaminase 1 (GLS1), is also highly expressed in tumorous tissues, and the GLS1 expression is correlated with a poorer survival rate in HNSCC patients. Under the treatment of the GLS1 selective inhibitor bis–2–(5–phenylacetamido–1,3,4–thiadiazol–2–yl)ethyl sulfide (BPTES), it was shown that a reduced glutamine consumption and HNSCC growth was detected, highlighting the significance of glutaminolysis in controlling the HNSCC malignancy [142].

Taken together, amino acid metabolic cues are important for HNSCCs, although much effort should be put in better identifying the significance of different amino acids in controlling the HNSCC physiology under the same experimental settings. In addition, based on the fact that different amino acid metabolisms crosstalk to each other, it becomes essential to apply isotope labeling methods to trace amino acid related metabolites, and their associations with phenotypic alterations and metabolic shifts. In this way, the metabolic plasticity of HNSCC cells could be more clearly illustrated, and key amino acid metabolic pathways could be uncovered.

## 5. Molecular Basis of Metabolic Regulations in HNSCCs

### 5.1. Non-Coding RNA (ncRNA) Mediated Control for HNSCC Metabolic Cues

Although the majority of cancer research focuses on investigating the roles of the protein-coding genes in controlling oncogenicity, the exon-coding strands only consist of 3% of the whole human genome [143]. The regulations of non-coding RNAs (ncRNAs) in physical and pathological conditions have remained largely unknown until recent years. Aberrant ncRNAs expression was found in a number of pathological conditions, such as Alzheimer’s disease, Down’s syndrome, Parkinson’s disease, neurodevelopmental disease, diabetes, leukemia, and various type of solid tumors [144]. As for tumourigenesis, ncRNAs regulate pathways for cancer initiation and progression in a tissue-specific manner [145]. Long non-coding RNA (LncRNA) and microRNA (miRNA), based on their length of nucleotides, were the most studied ncRNAs. LncRNAs are defined as ncRNAs that are more than 200 nucleotides long and associated with a range of biological conditions, including cell metabolism and immune response, as well as cancer development and progression [146]. In contrast, miRNAs are 19–22 nucleotides in length and involved in the regulation of gene expression by targeting mRNAs in order to cause a transcriptional repression or mRNA degradation [147,148].

Most ncRNA-mediated metabolic controls for tumorigenesis rely on regulatory cues derived by the surrounding environment. Cancer progression is highly dependent on the interactions between the tumor cell and the tumor microenvironment (TME); TME is comprised of various cell types, such as endothelial cells, cancer-associated fibroblasts (CAF), and immune cells, which may cross-talk with cancer cells via cytokines, growth factors, proteases or hormones [149]. It therefore may not be surprising that metabolic modulations in the tumor niche have considerable impacts in regulating oncogenicity. For example, immune checkpoint blockade therapies against CTLA-4, Program Death-1 (PD-1), and PD-L1 could restore glucose in the tumor microenvironment, resulting in an enhanced glycolytic activity in tumor infiltrating T lymphocytes (TILs) and IFN–production [150]. It was found that LncUCA1 promotes aerobic glycolysis by upregulating hexokinase 2 (HK2) and the mTOR-STAT3 pathway in bladder cancer [151]. LncHULC is highly expressed in hepatocellular carcinoma (HCC) patient tissues and positively correlated with the lipogenic gene ACSL1, which in turn controls intracellular triglycerides and cholesterol levels by increasing the miR-9-PPARA-ACSL1 axis [152]. Moreover, increased LncRNA LINC01234 promotes colon cancer cell growth and is associated with poorer survival via a regulation of the serine/glycine metabolism by serving as a competing endogenous RNA (ceRNA) for miR-642a [153]. The potential regulatory roles of ncRNAs in cancers such as lung, liver, gastric, colorectal, ovarian and prostate cancer have already been described elsewhere [146,154].

In HNSCC, the upregulation of LncCAF (FLJ22447) in cancer-associated fibroblasts (CAFs) upregulates cytokine IL-33, supports tumor growth and leads to a poor prognosis [155]. Using the TCGA database, it was found that LncZFAS1 serves as a stimulator in HNSCC and can be utilized as a potential tumor marker in clinic. On the molecular level, LncZFAS1 is associated with transcripts related to cell adhesion, cell differentiation, cell death, angiogenesis, oxidative stress response, as well as endothelial signal regulation. More recently, high levels of EGFR and PD-L1 were found in LncZFAS1-low patients, suggesting that a low expression of LncZFAS1 might be more responsive to anti-EGFR and anti-PDL1 therapies [156]. Furthermore, the upregulation of LncHIFCAR (long noncoding HIF-1αco-activating RNA, MIR31HG) in OSCC patients can also be a prognostic indicator via the direct interaction with Hypoxia inducible factor-1α (HIF-1α under hypoxia condition [157]). Interestingly, LncHIFCAR could induce pseudohypoxia by interacting with HIF-1 and a modulated hypoxia-induced glucose uptake and lactate production. In addition to LncZFAS1 and LncHIFCAR, hypoxia could also enhance the expression of another non-coding RNA, HAS2-AS1, in an HIF-1α dependent manner, and the increase of HAS2-AS1 contributes to hypoxia-regulated EMT and invasiveness in OSCC cells [158].

In addition to LncRNA, several studies supported the roles of miRNAs in orchestrating cross-talks between tumor cells and tumor stroma [159]. In HNSCC, miR-34a plays an inhibitory role by shaping a microenvironment favorable for tumor growth; a recent study demonstrated that miR-34a differentially modulate endothelial cell growth, migration and tube formation through a vascular endothelial growth factor (VEGF) mediated machinery in the microenvironments of pre-cancerous lesions and cancers [160]. Interestingly, CAF and CAF-derived exosomes contain lower miR-34a levels than normal fibroblasts do, as a lower miR-34a expression enhances the AKT, GSK3β, β-catenin, and snail signaling activity by targeting AXL [161]. Whilst there was still no evidence supporting the role of miR-34a in controlling the immune system in HNSCCs, nevertheless, in acute myeloid leukemia (AML), miR-34a could target PD-L1 to lessen PD-L1-mediated T cell apoptosis [162]. In contrast, miR-34a was reported as a potential invasive biomarker for the diagnosis of progressive pancreatic ductal adenocarcinoma (PDAC) [163], implying that miR-34-related regulatory mechanisms could vary in different cancers. Under hypoxia conditions, miR-210 can be induced in many cancer cells (breast, pancreatic, head and neck, lung, colon, and renal carcinoma) [164,165,166,167]. The HIF-1α-driven overexpression of glycolytic enzymes and miR-210 is coupled to the downregulation of its target iron-sulfur cluster assembly enzyme (ISCU) in oropharyngeal squamous cell carcinoma (OPSCC) [168]. Moreover, decreased miR-340 levels in OSCC cells led to an increased cell growth via targeting GLUT1 expression, which consequently resulted in an enhanced lactate secretion and glucose uptake [169]. As ncRNAs could potentially target multiple downstream molecular cues, a cooperative regulation between LncRNAs and miRNAs was also reported. It was shown that LncPCAT19 promotes the proliferation and poor prognosis of larynx carcinoma, and that the LncPCAT19-mediated regulation in laryngeal cancer cells occurred through the miR-182-mediated facilitation of the mitochondrial PDK4 expression [156].

Aside from the LncRNAs and miRNAs detected in the tumor or its microenvironment, the analysis for serological and salivary LncRNAs and miRNAs as potential diagnostic tools has recently been carried out [163,170,171,172]. It was found that LncRNAs HOXA11-AS, LINC00964 and MALAT-1 were upregulated in the plasma of HNSCC patients when compared to healthy controls, while the levels of miR-21 [173] and miR-146a [174] were significantly increased in plasma samples obtained from HNSCC patients. On the other hand, the numbers of LncRNAs, including MALAT-1, HOTAIR, NEAT-1, HULC, MEG-3 and UCA1, were abundant in both HNSCC tumor tissues and patients’ saliva [175] as two salivary miRNAs, miR-125a and miR-200a, were significantly decreased in OSCC patients compared to healthy subjects [176]. Interestingly, miR-21 is not only a plasma biomarker for HNSCC but also a salivary marker for the detection of early esophageal cancer [177,178]. Based on our findings, one of the oncogenic miRNAs, miR-31, was increased in both the plasma and saliva of the OSCC patients at all clinical stages when compared with normal subjects; the miR-31 level is abundantly detected in the saliva when compared to the plasma, suggesting that salivary miR-31 is a more sensitive marker for oral malignant transformation [179,180]. Additionally, in a 4NQO-induced oral cancer mouse model, the plasma and salivary miR-21, 31, 146a, and 184 expressions were all increased in progressive tongue cancers [181]. Taken together, these findings demonstrated that the LncRNA and/or miRNA levels in saliva and plasma could be used as an early, noninvasive, and rapid tool for the diagnosis of oral cancer. In order to gain a more comprehensive view of metabolic networks regulated in HNSCC development, using different databases such as TCGA or GEO datasets to define potential therapeutic ncRNAs could be an alternative for exploring new HNSCC-associated ncRNAs [182,183]. The significance of ncRNAs in controlling the HNSCC malignancy is summarized in Table 1.

### 5.2. Crosstalk of Tumors and Microenvironment in HNSCCs

**Figure 2:**
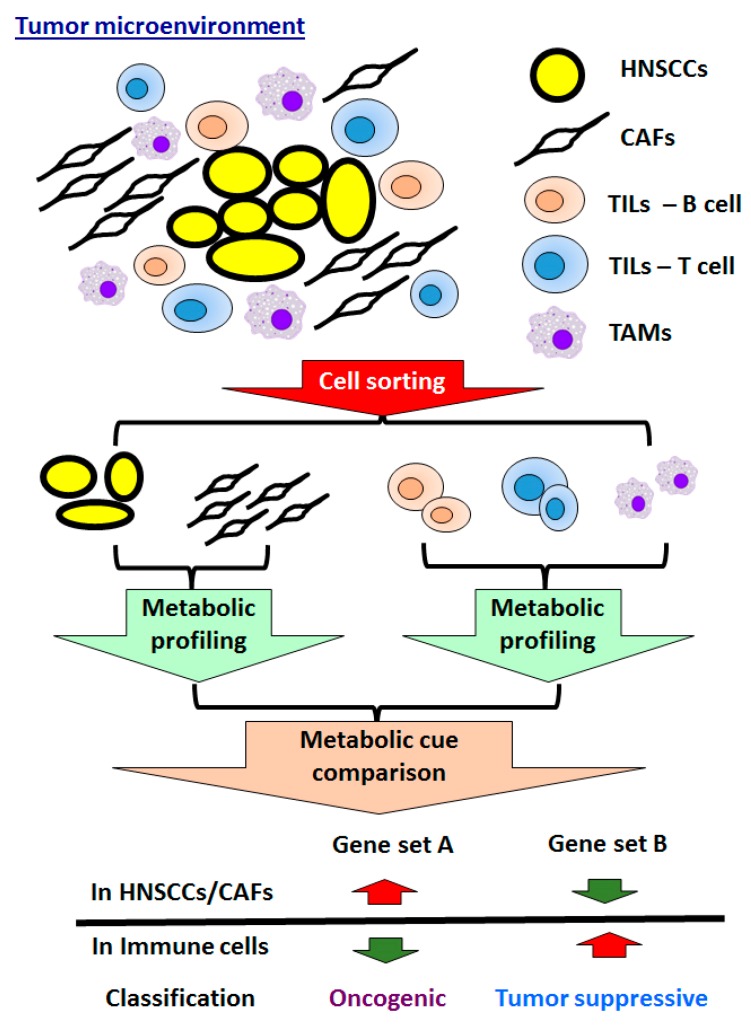
Exploring TME-associated tumor activators/inhibitors in HNSCCs. A comparison between the metabolic profiles of sorted tumor cells, CAFs and tumor-associated immune cells could define oncogenic or tumor suppressive metabolic molecules. The reciprocal expression between HNSCCs/CAFs and tumor-associated immune cells could be molecules of interest. CAFs: Cancer associated fibroblasts; TILs: Tumor infiltrating lymphocytes; and TAMs: Tumor associated macrophages.

Tumor cells rely on external nutrients for proliferation and survival, and it is therefore not surprising that the cancer milieu plays a crucial role in controlling tumourigenesis [184,185,186,187,188]. By using an immunohistochemistry analysis, an early focus in investigating the role of TME during cancer development was initiated by defining stromal-enriched proteins. For example, a number of studies reported that VEGF [189], Receptor for Advanced Glycation End products (RAGE) [190], Platelet-derived Growth Factor Receptor PDGFR) [191], Hepatocyte growth factor/c-met (HGF/HGFR) signaling [192,193,194], Tumor Growth Factor 1(TGF-1)-Throbospondin-1(THBS-1) axis [195], Notch signaling [196] and Wnt signaling [197] molecules serve as tumor stroma specific factors and have a great impact in regulating the HNSCC malignancy. Regarding the methodology, in order to better define the importance of stromal cells in modulating the HNSCC cell malignancy, a tumor-stroma co-culture system was developed [198]. Much effort was also spent to elucidate the significance of different cell populations detected in tumor stroma — mainly CAFs and tumor-associated immune cells, including tumor-associated macrophages (TAMs) and tumor-infiltrating lymphocytes (TILs) — in modulating HNSCC oncogenicity (Figure 2). The importance of another important environmental cue, hypoxia, in influencing the tumorous identification of HNSCC cells was also a research field of great interest. Last, very recent studies showed that tumor-secreted lactate and monocarboxylate transporters (MCTs) detected in tumor and stromal cells also play important roles in modulating HNSCC progression [199,200,201,202], suggesting that a better understanding of the metabolic competition between tumor cells and surrounding immune cells is essential for development of anti-HNSCC therapy [203].

#### 5.2.1. CAFs in HNSCCs

The first observation for determining the importance of CAFs in HNSCC was described by Rosenthal et al., showing that elevated TGF 1 was detected in the stromal counterpart of HNSCCs compared to normal mucosa [204]; the stroma-derived TGF 1 is important to the migration of Disseminated Tumor Cells (DTCs) [205]. Moreover, a co-culture of HNSCC/OSCC cells with CAFs could trigger an HNSCC cell proliferation, invasion and metastatic activity [206], as well as metabolic alteration [81]. The large-scale screening of secretome by CAFs was also carried out to more systemically identify stroma-specific molecules, and the results found that the epithelial-mesenchymal-transition (EMT) mediator S100 calcium-binding protein A4 (S100A4) serves as a potential therapeutic target for the CAFs-based treatment in HNSCCs [207]. Interestingly, a very recent study demonstrated that the crosstalk between OSCC cells and CAFs via the tumor microvesicles mediated “Reverse Warburg effect” could promote tumor cell motility and facilitate glycometabolic activity [208].

#### 5.2.2. Immune Cells in HNSCCs

In theory, the immune system could protect cells from attacks by microorganisms and pathological cells including tumor cells, in order to maintain homeostasis in the body. Although TILs could be found in clinic HNSCC tumor tissues, the elevated expression of the death-associated domains Fas and Fas ligand (FasL) was also detected [209], indicating that immune cells in TME might be suppressive and functionally exhausted. Different immune cell populations were defined in HNSCC TME, and they execute immune suppression via various molecules, mainly cytokines, to promote tumor growth. For instance, CD4+/CD25high/Foxp3+ Treg cells secreted IL-10/TGFβ1 to inhibit TME [210]; Neutropolin-1-/- Treg cells produced Interferon-γ triggering Neutropolin-1+/+ Treg cell fragility to modulate TME immune activity [211]; HNSCC cells target Th17+ cells for an increased Cox2 expression, resulting in greater tumor cell growth [212]; TAMs enhanced HNSCC invasiveness and lymph node metastasis via IL-33 and the CCR4/CCL22 axis, respectively [213,214]; and immunosuppressive adenosine (ADO) is reduced in HNSCC patients compared with healthy control subjects [215]. With great attention paid to immune checkpoint molecules such as PD-1 and its ligand PDL1 in tumor immunology [216], several studies were conducted to test whether a blockade of immunosuppressive TME could rescue anti-tumor activity in HNSCC patients. The blockade of PD-1/SHP2 could restore a robust Th1 immunity and thereby reverse immunosuppression in TME [217]. Stromal IL-33 is important for the suppression of immune activity and is correlated with a poorer prognosis in HNSCC patients [218]. It is worth noting that scores of different immune cell subpopulations (dendritic cells (DCs), CD4+/CD8+ T cells, IL-12Rβ2+ TILs [219,220,221] and CD47+ macrophages [222]) predict differential clinical outcomes in various studies, even though a very recent meta-analysis showed that a high PDL1 expression did not correlate with a poorer prognosis for OSCC patients [223]. The discrepant findings indicated that a more complex molecular regulation for modulating the immune activity remains to be explored.

#### 5.2.3. Hypoxia/Vascular Signals in HNSCCs

Hypoxia stimulates a complex oncogenic alteration in tumors [224]. Oxygen deprivation in cancerous tissues often leads to an advanced but dysfunctional vascularization to support tumor growth and invasion [225]. In HNSCCs, it was long found that a very low oxygen pressure (< 10 mmHg) was detected, compared to normal tissues (~43 mmHg) [226]. At the molecular level, the HIF-1α/MIF and NF-κB/IL-6 axes contributed to the recruitment of CD11b+Gr-1+ immunosuppressive myeloid cells and promoted angiogenesis and tumor invasion [227]. Hypoxic HNSCC tumors could secret miR-21 containing exosomes into normoxic cells to enhance metastatic activity [228]. Under hypoxic conditions, vascular distant HNSCC cells displayed a lower expression of the Epidermal Growth Factor Receptor (EGFRs), which may underlie anti-EGFR therapy resistance [229]. A very recent study demonstrated that primary hypoxic TME gives rise to a population of dormant DTCs that evade therapy and may contribute to disease relapse and poor prognosis [230]. On a molecular level, it was shown that the oncogenic p38-MAPK signaling pathway could facilitate angiogenesis and lymphangiogenesis in HNSCCs [231].

## 6. Conclusions and Future Perspectives

**Figure 3:**
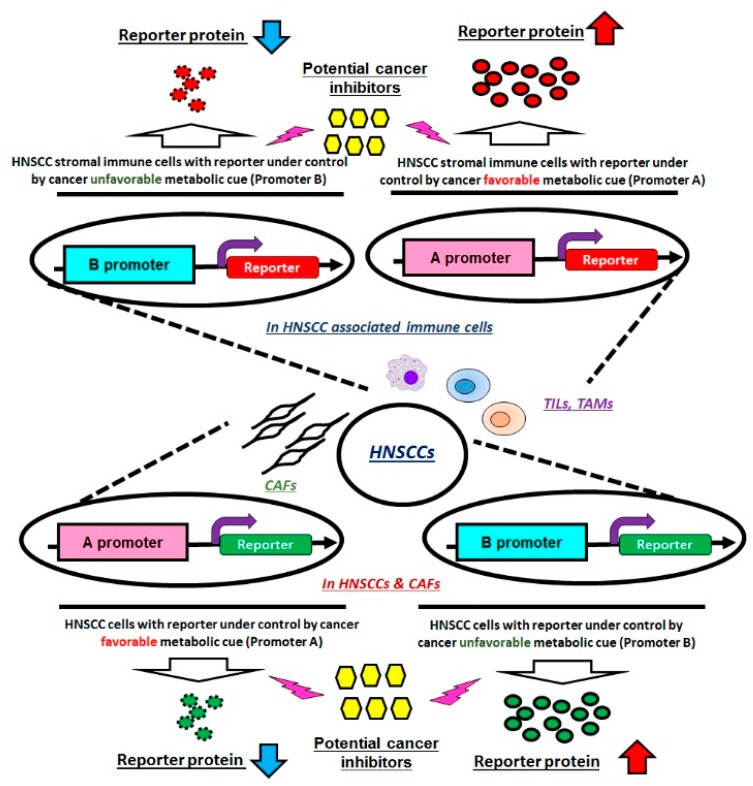
The hypothetic experimental design for novel metabolism-mediated anti-cancer drug screening. HNSCC and CAF cells containing reporters driven under a cancer favorable gene promoter (A promoter) and cancer unfavorable gene promoter (B promoter) could be established, while the same plasmid cassettes could also be introduced into tumor-associated immune cells. The system could be then utilized to screen potential cancer inhibitors via the detection of reporter alterations. CAFs: Cancer associated fibroblasts; TILs: Tumor infiltrating lymphocytes; and TAMs: Tumor associated macrophages.

Cellular metabolism is highly dynamic, and the regulation between environmental and intracellular metabolic cues is closely intertwined. In order to translate the current knowledge about cancer metabolism for the development of clinical therapeutic schemes, several points should be addressed. First, the effects of using combinational therapy of conventional chemo/radiotherapy plus metabolic modulators in treating HNSCCs were rarely examined. While working mechanisms vary for conventional therapies (mainly targeting cell growth) and metabolism-mediated anti-cancer agents, it would be interesting to determine whether combinational therapies could be superior to single treatments. Second, in order to manipulate tumorous metabolic cues in a broader manner, an ideal platform for screening potential reagents that suppress cancerous metabolic molecules and simultaneously upregulate less active metabolic factors in cancer cells would be required. Last, it is likely that HNSCC tumors and stromal cells exhibit reciprocal metabolic cues that could be important in helping cancer cells escape from immune attacks and, at the same time, in exhausting immune cells in TME. It therefore becomes essential to define metabolic molecules that display opposite expression pattern in tumors/CAFs and tumor-associated immune cells on a single-cell population level. In this way, the candidate compound(s) could be more efficiently suppressing the malignancy and stimulating immune cell activity (Figure 3).

## Figures and Tables

**Table 1:** Summary of the ncRNA-mediated regulations for the HNSCC metabolism.

Cancer Type	ncRNA	Regulating Target/Role	Molecular Alterations	Reference
Bladder cancer	LncUCA1	glucose metabolism	hexokinase 2 (HK2)↑, mTOR-STAT3↑	[151]
Hepatocellular carcinoma	LncHULC	lipid metabolism	ACSL1↑	[152]
Colon cancer	LncRNA LINC01234	amino acid metabolism	miR-642a↓	[153]
Head and neck cancer	LncCAF (FLJ22447)	oncogenic	IL-33↑	[155]
Head and neck cancer	LncZFAS1	tumor suppressive	EGFR↓, PD-L1↓	[156]
Head and neck cancer	LncHIFCAR (MIR31HG)	glucose metabolism	HIF-1↑	[157]
Head and neck cancer	ncHAS2-AS1	hypoxia		[158]
Larynx carcinoma	LncPCAT19	mitochondria	miR-182↑	[156]
Head and neck cancer	miR-34a	tumor suppressive	VEGF↓, AXL↓	[160,161]
Acute myeloid leukemia	miR-34a		PD-L1↓	[162]
Pancreatic ductal adenocarcinoma	miR-34a			[163]
Oropharyngeal squamous cell carcinoma	miR-210		ISCU↓	[168]
Head and neck cancer	miR-340	glucose metabolism	GLUT1↑	[169]
Head and neck cancer	miR-21, LncRNAs HOXA11-AS, LINC00964 and MALAT-1	plasma biomarker		[173]
Head and neck cancer	miR-146a	plasma biomarker		[174]
Head and neck cancer	MALAT-1, HOTAIR, NEAT-1, HULC, MEG-3 and UCA1	oncogenic		[175]
Head and neck cancer	miR-125a and miR-200a	salivary marker		[176]
Esophageal cancer	miR-21	salivary marker		[177,178]
Head and neck cancer	miR-31	plasma and salivary biomarker		[179,180]
